# A Heuristic Ranking Approach on Capacity Benefit Margin Determination Using Pareto-Based Evolutionary Programming Technique

**DOI:** 10.1155/2015/731013

**Published:** 2015-03-23

**Authors:** Muhammad Murtadha Othman, Nurulazmi Abd Rahman, Ismail Musirin, Mahmud Fotuhi-Firuzabad, Abbas Rajabi-Ghahnavieh

**Affiliations:** ^1^Committee of Research (CORE), Advanced Computing & Communication (ACC), Universiti Teknologi MARA, 40450 Shah Alam, Selangor, Malaysia; ^2^Faculty of Electrical Engineering, Universiti Teknologi MARA, 40450 Shah Alam, Selangor, Malaysia; ^3^Engineering Centre, University Malaysia Perlis, Kampus Kubang Gajah, 02600 Arau, Perlis, Malaysia; ^4^Centre of Excellence in Power System Management and Control, Electrical Engineering Department, Sharif University of Technology, Tehran 11365-11155, Iran; ^5^Department of Energy Engineering, Sharif University of Technology, Tehran 11365-11155, Iran

## Abstract

This paper introduces a novel multiobjective approach for capacity benefit margin (CBM) assessment taking into account tie-line reliability of interconnected systems. CBM is the imperative information utilized as a reference by the load-serving entities (LSE) to estimate a certain margin of transfer capability so that a reliable access to generation through interconnected system could be attained. A new Pareto-based evolutionary programming (EP) technique is used to perform a simultaneous determination of CBM for all areas of the interconnected system. The selection of CBM at the Pareto optimal front is proposed to be performed by referring to a heuristic ranking index that takes into account system loss of load expectation (LOLE) in various conditions. Eventually, the power transfer based available transfer capability (ATC) is determined by considering the firm and nonfirm transfers of CBM. A comprehensive set of numerical studies are conducted on the modified IEEE-RTS79 and the performance of the proposed method is numerically investigated in detail. The main advantage of the proposed technique is in terms of flexibility offered to an independent system operator in selecting an appropriate solution of CBM simultaneously for all areas.

## 1. Introduction

In a deregulated power system environment, electricity is considered as a commodity that can be traded in a free market where the generators and loads participated. The transition to a new structure of electricity market is to ensure the quality and efficient production of electrical energy that can be offered at a lower electricity price as well as maximizing the utilization of generation and transmission facilities [1, 2]. Hence, it is important for the independent system operator (ISO) to calculate and provide the information of available transfer capability (ATC) associated with the transfer paths to the open access same-time information system (OASIS) so that electricity market could be conducted in an effective manner [3, 4]. ATC is defined as the maximum amount of power that can be transferred from a selling area to a buying area without jeopardizing a system security [5]. ATC can be calculated as the total transfer capability (TTC) reduced by the transmission reliability margin (TRM), capacity benefit margin (CBM), and existing transmission commitment (ETC). CBM is one of the main components considered in the ATC calculation and is defined as the amount of transfer capability reserved by load-serving entities, which is anticipated to be used in cases of generation deficiency [5–9]. Inaccurate determination of CBM may result in either underestimation or overestimation of the ATC. Underestimating the ATC value possibility will cause an ineffective use in the transmission facility, while overestimating the ATC value will threaten a power system security [3, 7].

So far, several methods have been proposed to determine CBM [10–19]. The basic method used to compute the CBM for each area of an interconnected system is based on trial and error [10], by prescribing 5% of the maximum transfer capability [11] or the CBM value is specified as zero [12, 13]. Reference [14] has proposed an analytic model used for multiarea generation reliability assessment and then applied into the sequential quadratic programming (SQP) for determining the CBM values considering the loss of load expectation (LOLE) as the system reliability criterion. Rajathy et al. [15] use the differential evolution and Monte Carlo techniques to determine the CBM. A method that has been proposed in [16] is used to determine the CBM for each area of an interconnected system using the evolutionary programming (EP) as an accelerated search technique. Furthermore, CBM determination is formulated as an optimization problem which is solved by using the particle swarm optimization (PSO) technique [17, 18]. In order to provide a set of choices for different cases, three methods have been proposed in [17, 18] which will provide different values of CBM. It is observed that the existing CBM calculations do not provide adequate flexibility for the ISO to select a CBM value in accordance with system requirements [10–19]. In addition, tie-line availability is an influential factor which has an effect on the reliability of an interconnected system followed by the value of CBM. This imperative factor has been taken into account for CBM calculation in [19].

A novel multiobjective based optimization approach is presented in this paper to determine several optimum values of CBM using the Pareto-based EP technique that takes into account the tie-line reliability of an interconnected system. The proposed Pareto-based EP technique has several advantages compared to the methodology previously presented in [16] and it provides the ISO with several choices of optimum CBM values. The multiobjective function of EP technique is referred to as the transfer capability margin of CBM for all areas with LOLE less than a specified value at initial condition. Moreover, the CBMs of all areas are obtained simultaneously at every execution of the proposed technique. The first order sensitivity with modified Gaussian formulation is used as a new mutation technique to enhance the EP performance in searching for a new population at global maximum domain with less computational time. Then, the Pareto optimal front approach is used to select several optimal solutions of CBM values using the ranking index of total LOLE and total difference of LOLE. A modified IEEE-RTS79 is used as the numerical test bed to verify effectiveness of the proposed method in providing the solutions of CBMs [17]. The robustness of the proposed method in CBM determination is compared with that of the basic methodology used for the CBM calculation [17]. Performance comparison has also been performed which investigates the effect of tie-line reliability included in the CBM determination. Finally, the significance of CBM considered as firm and nonfirm transfers can be observed through its impact on the ATC determination.

## 2. Multiobjective Functions of Capacity Benefit Margins Determination

A process involved in the Pareto-based EP technique used for determining the multiobjective function of CBMs is described as follows.

*Step (a)*. Establish a solved base case power flow solution.

*Step (b).* Determine the LOLE for each area of the interconnected system at the base case condition.

*Step (c).* Identify the assisting areas with LOLE less than the specified value, *ξ* (e.g., 2.4 hrs/yr). It signifies that these areas conserve a certain amount of reserve generating capacity that could be used to compensate for the generation deficiency which may occur in the assisted area. LOLE associated with the assisted area is usually greater than *ξ*. It is important to mention that the assisting and assisted areas are the terms used to signify the direction of power transfer based CBM (CBM_asg_^Pareto^) and this is different from the selling and buying areas which are the terms used to signify the direction of power transfer based ATC.

*Step (d).* Identify the assisted area with the largest LOLE above *ξ*.

*Step (e).* Determine the parent or initial population for each assisting area with LOLE below *ξ*. Equation (1) is used to generate the individuals *x*par_*m*,asg_, for parent or initial population using uniform random distribution. The determination of *x*par_*m*,asg_ is based on either total rating of all tie-lines connecting between the assisting and assisted areas, PLIt_asg_, or the total reserve generating capacity of the assisting area, DPGt_asg_. The *x*par_*m*,asg_ is determined based on the former condition when DPGt_asg_ exceeds the PLIt_asg_. This means that tie-lines are the constraining factors for power transfer based CBM and, thus, *x*par_*m*,asg_ are generated randomly based on PLIt_asg_. The latter condition is used to determine *x*par_*m*,asg_ when DPGt_asg_ is less than PLIt_asg_. Each individual, *x*par, is considered as an external generating capacity, PG_Ext_, or CBM, which is  provided by the assisting area to support generating capacity deficiency in the assisted area having the highest LOLE:

(1) xparm,asg=randmDPGtasg,if  DPGtasg<PLItasg,randmPLItasg,if  DPGtasg>PLItasg,

where

(2) DPGtasg=PGtasg−PLtasg,PLItasg=∑l=1LPLIlasg.

CBM_*m*,asg_ or *x*par_*m*,asg_ is the CBM in the case of transfer from assisting area to assisted area; PGt is the total generating capacity; PLt is the total peak load; PLI is the tie-line rating; *L* is the total number of tie-lines; *m* is 1,2, 3,…, pop; asg is 1,2, 3,…, *N*asg; pop is the population size; and *N*asg is the total number of assisting areas.

*Step (f).* Calculate a new total generation capacity, new PGt_*m*,asg_, for each assisting area according to CBM or *x*par_*m*,asg_ as given in (3) and (4). The generating capacity of the assisting area is reduced as it is partially assigned to the assisted area. The new generating capacity for each bus *g* of the assisting area new PGt_*m*,asg_ is obtained based on the ratio of generating capacity as

(3) new PGtm,asg=∑g=1NGnew PGgm,asg,

where

(4) new PGgm,asg=PGgasg−PGgasg∑g=1NGPGgasg×xparm,asg.

PG is the generating capacity and NG is the total number of generator buses.

*Step (g).* Determine the LOLE for each assisting area (LOLE_*m*,asg_) considering the new PGt_*m*,asg_, hourly peak load, and cumulative probability of generation capacity outage (PC(*C*_*s*_)) as discussed in [19].

*Step (h).* Determine a new total generation capacity, new PGt_*m*,asd=1_, for an assisted area with the largest LOLE above *ξ* using (5) and (6). In (6), apportionment of the total *x*par_*m*,asg_ or total CBM_*m*,asg_ to each generator is performed based on the ratio of generating capacity and total generating capacity of an assisted area. For an assisted area, there are pop number of individuals for the size of new total generating capacity, new PGt_*m*,asd=1_,

(5) new PGtm,asd=1=∑g=1NGnew PGgm,asd=1,

where

(6) new PGgm,asd=1=PGgasd=1+PGgasd=1∑g=1NGPGgasd=1∑asg=1Nasgxparm,asg,

where asd is the number of assisted areas, 1.

*Step (i).* Calculate the fitness value (*f*_*m*_), that is, LOLE_*m*,asd=1_ as discussed in [19]. *f*_*m*_ is an important parameter used to assist the determination of a new *x*par_*m*,asg_ and the convergence criteria for the optimization process. This will be explained thoroughly in the following steps. *f*_*m*_ or LOLE_*m*,asd=1_ is calculated by taking into account the increased amount of new PGt_*m*,asd=1_ obtained in Step (h).

*Step (j).* Perform the mutation to obtain an offspring for each assisting area with LOLE less than *ξ*. In the proposed mutation approach, the modified Gaussian technique is used to improve the capability of global maximum search of a new population with less computational time [16]. This technique is suitable in solving the optimization problems in which considerable discrepancy does exist among the individual values. Each offspring comprising new individuals, *xoff*⁡_*m*,asg_, is originated from *x*par_*m*,asg_. The new individuals, *xoff*⁡_*m*,asg_, are obtained using a new mutation technique that incorporates the first order sensitivity, ∂*x*par_asg_/∂*N*(*f*, *ξ*, *σ*), and the modified Gaussian formulation, *N*(*f*_*m*_, *ξ*, *σ*), as expressed in (7). The value of *xoff*⁡_*m*,asg_ is varied in accordance with the changes in *f*_*m*_ to the estimated LOLE limit, *ξ*. Consider

(7) xoff⁡m,asg=xparm,asg+∂xparasg∂Nf,ξ·σ1−Nfm,ξ,σ,

where

(8) ∂xparasg∂Nf,ξ,σ=max⁡⁡xparasg−min⁡xparasgmax⁡⁡Nf,ξ,σ−min⁡⁡Nf,ξ,σ,Nfm,ξ,σ=e−fm−ξ2/2σ2,

where max⁡*x*par_asg_ and min⁡*x*par_asg_ are the maximum and minimum values of *x*par_*m*,asg_ for every assisting area, respectively; max⁡*N*(*f*, *ξ*, *σ*) and min⁡*N*(*f*, *ξ*, *σ*) are the maximum and minimum values of *N*(*f*_*m*_, *ξ*, *σ*), respectively; and *σ* or *f*_max⁡_ is the maximum value of fitness, *f*_*m*_ or LOLE_*m*,asd=1_.

The first order sensitivity is used to overcome the impediment of local maxima or minima which normally occurs in the case of large *f*_*m*_. Hence, robustness in searching for the global maxima or minima can easily be guaranteed by using the new mutation technique.

*Step (k).* Perform Steps (h) and (i) to determine *f*_*m*_ or LOLE_*m*,asd=1_ in relation to a new value of new PGt_*m*,asd=1_ obtained according to (5) considering *xoff*⁡_*m*,asg_. This implies that the *x*par_*m*,asg_ in (6) has been replaced by *xoff*⁡_*m*,asg_, yielding to a new value of new PGt_*m*,asd=1_. Apart from the new PGt_*m*,asd=1_ obtained based on *xoff*⁡_*m*,asg_, determination of LOLE_*m*,asd=1_ also requires several other parameters such as the hourly peak load and new cumulative probability of the generation capacity outage (PC(*C*_*s*_)) as discussed in [19].

*Step (l).* Perform pairwise comparison to determine the next generation of population comprising the best individuals selected from *xoff*⁡_*m*,asg_ and *x*par_*m*,asg_. For each assisting area, *f*_*m*_ or LOLE_*m*,asd=1_ has been used as a reference for selecting the best individuals as the next generation of *x*par_*m*,asg_. In this case, *f*_*m*_ for *x*par_*m*,asg_ and *xoff*⁡_*m*,asg_ are obtained from Steps (h) and (j), respectively. The concept of selection is elucidated in terms of the formulation given in (9). Otherwise, when the total number of chosen individuals is not adequate for population size, pop, then the offspring, *xoff*⁡_*m*,asg_, is selected as the next generation of *x*par_*m*,asg_ as illustrated in

(9) xselm,asg=xoff⁡m=1,asgfm=1xoff⁡m=1,asg<ξ⋮⋮xoff⁡m,asgfmxoff⁡m,asg<ξ⋯⋯⋯⋯⋯⋯⋯⋯⋯⋯⋯⋯⋯xparm=1,asgfm=1xparm=1,asg<ξ⋮⋮xparm,asgfmxparm,asg<ξ,

(10) xparm,asg=xselm,asg,if sizexselm,asg≥pop,xoff⁡m,asg,if sizexselm,asg<pop,

where *x*sel_*m*,asg_ is the best individuals selected from *xoff*⁡_*m*,asg_ and *x*par_*m*,asg_ having *f*_*m*_ < *ξ*; *f*_*m*_(*xoff*⁡_*m*,asg_) is the *f*_*m*_ corresponding to the *m*th value of individual *xoff*⁡_*m*,asg_; *f*_*m*_(*x*par_*m*,asg_) is the *f*_*m*_ corresponding to the *m*th value of individual *x*par_*m*,asg_; and size (*x*sel_*m*,asg_) is the size of *x*sel_*m*,asg_.

*Step (m).* The convergence criteria for the EP optimization process is achieved when the mismatch between maximum fitness, *f*_max⁡_, and minimum fitness, *f*_min⁡_, is within a specified range, *ε*. *f*_max⁡_ and *f*_min⁡_ are the maximum and minimum values of *f*_*m*_, respectively, obtained based on the *x*par_*m*,asg_ in Step (l):

(11) fmax⁡−fmin⁡≤ε,

where *f*_min⁡_ is the minimum value of *f*_*m*_ or LOLE_*m*,asd=1_ and *ε* is the desired accuracy, 0.1 for an example [16].

Go to Step (f) for the next generation of EP optimization process when the mismatch does not reach to the desired level and the new value of *x*par_*m*,asg_ obtained in Step (l) will be used to calculate a new PGt_*m*,asg_ in Step (f). Otherwise, proceed to Step (n) once the mismatch has reached the predetermined limit *ε*.

*Step (n).* Record the optimized multiobjective function of CBM_asg_ for the transfer case from assisting areas to an assisted area. The optimized multiobjective CBM_asg_ will be recorded at the last iteration of the optimization process. The CBM_asg_ is obtained as the average value of *x*par_*m*,asg_ or CBM_*m*,asg_ associated with the assisting area previously calculated in Step (l). This implies that the CBM_asg_ is calculated through (12). Hence, the multiobjective function (M.O.F) comprising several optimized CBM_asg_ for the case of power transferred from the assisting areas can be expressed by (13). Then, LOLE_asg_ is computed based on the CBM allocated for each assisting area, CBM_*m*,asg_, as discussed in [19]. Consider

(12) CBMasg=μxparm,asg=μCBMm,asg,

(13) M.O.F=CBMasg=1,CBMasg=2,…,CBMasg=Nasg.

Therefore, CBM_asd_ for an assisted area is calculated by summing the optimum amount of CBM_asg_ transferred from all the assisting areas as given in

(14) CBMasd=1=∑asg=1NasgCBMasg.

**Figure 1:**
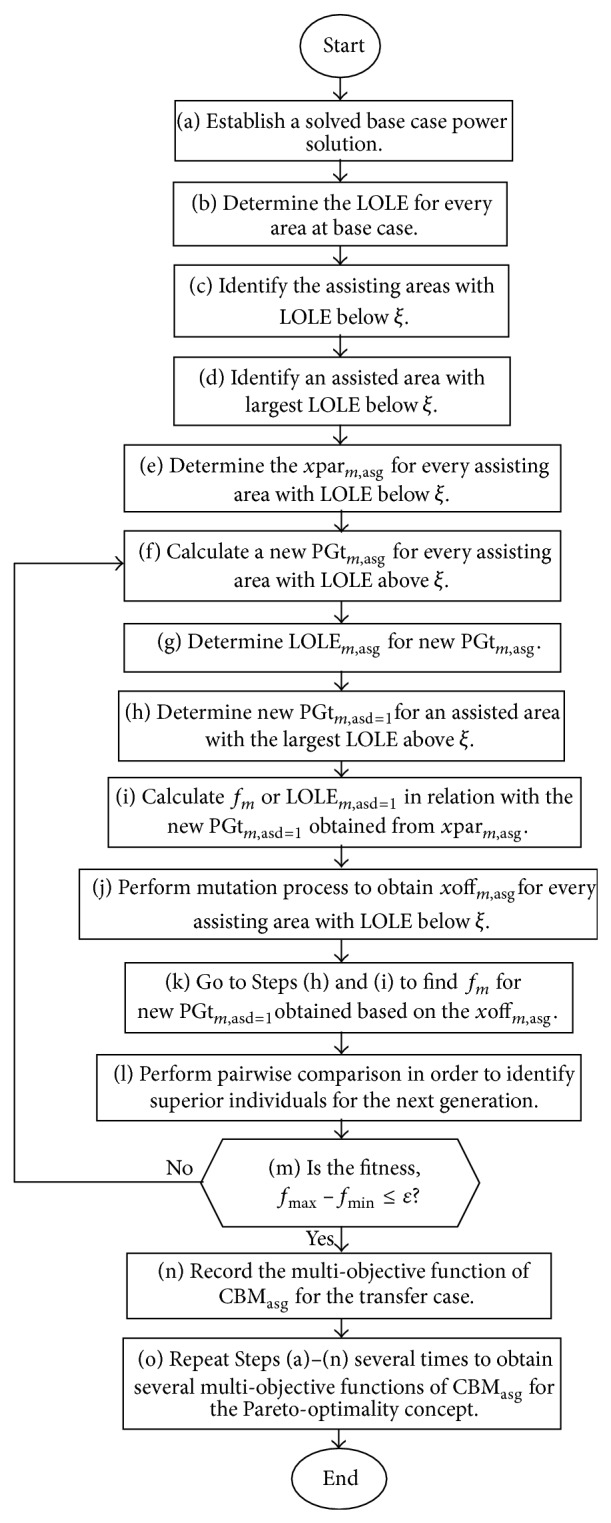
Proposed EP technique to determine several multiobjective functions of CBMs.

*Step (o).* Repeat Steps (a)–(n) several times in order to obtain numerous optimal solutions of multiobjective CBM_asg_. These results will be applied into the Pareto optimal concept in such a way to find several superior multiobjective CBM_asg_.  Figure 1 presents the flowchart of the proposed EP optimization technique used to determine several multiobjective functions of CBMs.

## 3. Ranking Index in the Pareto Optimality Concept for the Best Selection of Optimal Multiobjective Capacity Benefit Margins

**Figure 2:**
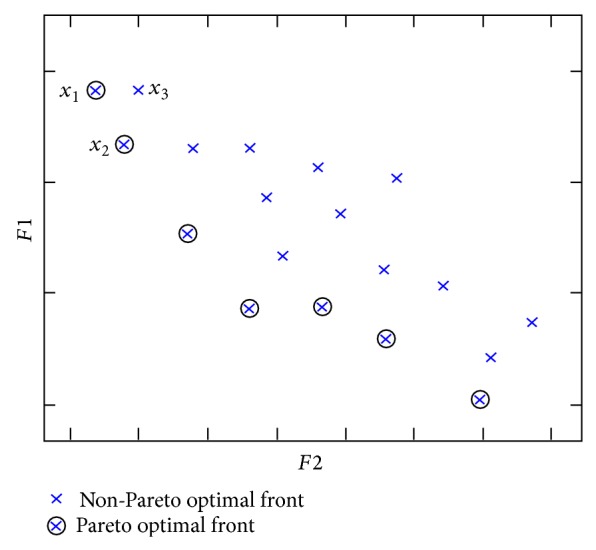
Pareto and non-Pareto optimal fronts for the multiobjective function CBM_asg_.

Pareto optimality is a concept that has been commonly used to select several optimal solutions of the multiobjective CBM_asg_ designated as multiobjective CBM_asg_^Pareto^. This implies that the concept of Pareto does not provide a single solution that can be considered as the global optima for a problem related to the multiobjective CBM_asg_. This is important to the ISO since it will provide flexibility to select the optimal as well as the most inexpensive result of multiobjective CBM_asg_^Pareto^. These inexpensive results usually fall under the cluster of the Pareto optimal front. However, it is not worthy to select an expensive optimal result of multiobjective CBM_asg_ and this type of solution is usually categorized under the cluster of non-Pareto optimal.  Figure 2 shows an example elucidating two clusters of the Pareto optimal concept. In Figure 2, *F*1 represents the axis plane of CBM_asg=1_ solution for the transfer case from assisting area 3 to assisted area 1. *F*2 is the axis plane of CBM_asg=2_ solution for the transfer case from assisting area 2 to assisted area 1.

The EP optimization technique is performed several times in order to provide numerous optimal solutions of CBM_asg_. In addition, solution *x* is the intersection point for the two CBM_asg_ results. The solutions *x* marked with a circle represent the cluster of Pareto optimal front. Usually, the best optimal solution of CBM_asg_, so-called CBM_asg_^Pareto^, is selected from the cluster of Pareto optimal front. Solutions *x* marked with × represent the cluster of non-Pareto optimal front which do not have the best optimal solution of CBM_asg_ due to their expensive multiobjective function. For instance, this can be observed through the comparison between *x*_1_ and *x*_3_, which have the same CBM_asg=1_ value for the *F*1 axis, that is, the transfer case from assisting area 3 to assisted area 1. However, by referring to the *F*2 axis, that is, the transfer case from assisting area 2 to assisted area 1, *x*_3_ yields to an expensive CBM_asg=2_ value compared to *x*_1_. Thus, *x*_3_ and *x*_1_ are optimal solutions of multiobjective CBM_asg_ which can be categorized under the non-Pareto and Pareto optimal fronts, respectively.

Theoretically, the Pareto optimal front can be defined as the solution *x* that is not dominated by any other feasible solutions *x* [20]. If the domination operator is labeled “≻,” the Pareto optimal concept can be described through the following criteria and this is referring to Figure 2.

- *x*_1_≻*x*_3_ and *x*_2_≻*x*_3_. Hence, the *x*_3_ solution is said to be dominated or a non-Pareto optimal front solution.
- *x*_1_≻*x*_2_ and *x*_2_≻*x*_1_. Hence, the *x*_1_ and *x*_2_ solutions are said to be nondominated or Pareto optimal front solution.

The aforementioned criteria can also be used to determine the Pareto optimal front for a multiobjective function which has more than two transfer case solutions of CBM_asg_.

Furthermore, the selection of CBM_asg_^Pareto^ will be performed by the ISO according to the ranking index of either total LOLE or total LOLE difference. The proposed method has the advantage of introducing CBM_asg_^Pareto^ which will also provide the optimum results of LOLE and LOLE difference located at the Pareto optimal front cluster. In the initial selection based on the ranking index of total LOLE, CBM_asg_^Pareto^ is arranged according to the ranking index of total LOLE sorted in an ascending order. Then, the CBM_asg_^Pareto^ is selected in accordance with the ranking index of total LOLE as shown in

(15) CBMasgPareto∈Ranktotal LOLE,

where

(16) total LOLE=∑asg=1NasgLOLEasg.

Equation (15) shows that CBM_asg_^Pareto^ is selected based on the ranking index of reliability or total LOLE in the assisting areas.

In the subsequent selection based on the ranking index of total LOLE difference, CBM_asg_^Pareto^ is arranged according to the ranking index of total LOLE difference sorted in an ascending order. Then, the ranking index of total LOLE difference is used to select CBM_asg_^Pareto^. This is illustrated in

(17) CBMasgPareto∈Ranktotal ΔLOLE,

where

(18) total ΔLOLE=∑asg=1NasgLOLEasg−LOLEasgo,

where LOLE_asg_^*o*^ is the LOLE at the base case condition of each assisting area.

Finally, the selected CBM_asg_^Pareto^ will be taken into account as firm and nonfirm transfer margins in the ATC determination.

## 4. Firm and Nonfirm Available Transfer Capability Determination

This section discusses the ATC determination that takes into account each optimum CBM_asg_^Pareto^ value selected by referring to the ranking index of total LOLE and total LOLE difference. The proposed method uses the iterative power flow solutions to determine ATC by taking into account CBM_asg_^Pareto^ for the transfer case from an assisting area to an assisted area [21]. Basically, the determination of ATC considering CBM_asg_^Pareto^ requires an iterative power flow solution to be performed at every increase of generation capacity and load at the respective selling and buying areas until one of the system constraints is met. This method is used to determine ATC considering CBM_asg_^Pareto^ for the next case of power transfer. It is important to note that two approaches are available to calculate ATC taking into account CBM_asg_^Pareto^ as firm or nonfirm transfer. In the former approach, the assisting and assisted areas are experiencing changes in total generation capacity according to the firm transfer of CBM_asg_^Pareto^, whereas, in the latter approach, ATC is determined as the total transfer capability, TTC, reduced by CBM_asg_^Pareto^. The procedure for both approaches discussed in this paper are implemented as follows.

*Step (a).* Establish a solved base power flow solution.

*Step (b).* Specify the selling and buying areas for a power transfer.

*Step (c).* Proceed to Step (e) if CBM_asg_^Pareto^ is considered to be a nonfirm transfer. Otherwise, adjust the generation outputs according to CBM_asg_^Pareto^ for all areas. The modification of generation outputs in assisted area and assisting area is done by using (19) and (20), respectively,

(19) $$\begin{array}{c} {\text{new}\;\text{PG}}_{g}^{\text{asd}=1}={\text{PG}}_{g}^{\text{asd}=1}-\frac{{\text{PG}}_{g}^{\text{asd}=1}}{\sum_{g=1}^{\text{NG}}{\text{PG}}_{g}^{\text{asd}=1}}\overset{N\text{asg}}{\underset{\text{asg}=1}{\sum }}{\text{CBM}}_{\text{asg}}^{\text{Pareto}}, \end{array}$$

(20) $$\begin{array}{c} \text{new}\;{\text{PG}}_{g}^{\text{asg}}={\text{PG}}_{g}^{\text{asg}}+\frac{{\text{PG}}_{g}^{\text{asg}}}{\sum_{g=1}^{\text{NG}}{\text{PG}}_{g}^{\text{asg}}}{\text{CBM}}_{\text{asg}}^{\text{Pareto}}. \end{array}$$

Notice that (19) and (20) may cause the assisting area to transfer its reverse generation capacity (CBM_asg_^Pareto^) for compensating the generation deficiency which may occur in the assisted area. This is different from what has been dealt previously with, with (4) and (6) whereby the generating capacity of an assisting area and assisted area is decreased and increased, respectively, in order to identify the amount of generation capacity reserved for the CBM so that LOLE will be less than *ξ*.

*Step (d).* Perform the power flow solution to allow an assisting area to transfer power based CBM_asg_^Pareto^ required for compensating the generation deficiency occurring in the assisted area. 

*Step (e).* Simultaneously, increase the power injection and extraction at the selling and buying areas, respectively, until either one of the line flows or voltage constraints is met through the load flow solution. The lower and upper voltage limits are considered to be 0.90 and 1.10 p.u., respectively. The injected power is referring to the increase of generation capacity in a selling area resulting in a power transfer which will be extracted by the load increased in a buying area. The maximum power transfer so-called TTC is acquired once the increased power flow solution has met one of the system constraints as mentioned previously.

*Step (f).* Calculate the ATC at three different cases of TTC determined in Step (e). In conjunction with the TTC^*o*^ for the first case, the ATC at base case condition is obtained by employing (21) which does not require the execution of Steps (c) and (d):

(21) ATCo=TTCo−ETC,

where TTC^*o*^ is the total transfer capability or the maximum power transfer at base case condition obtained and ETC is the existing transmission commitment or base case load flow solution considering system components variations.

With regard to the TTC^*o*^ and CBM_asg_^Pareto^ for the second case, (22) is used to calculate ATC taking into account nonfirm transfer of CBM:

(22) ATCnonfirm=TTCo−CBMasgPareto−ETC.

By referring to TTC|_CBM_asg_^Pareto^_ given for the third case, the CBM is taken as a firm transfer for ATC determination and the associated formulation is introduced through

(23) $$\begin{array}{c} {\text{ATC}}_{\text{firm}}={\left.\text{TTC}\right|}_{{\text{CBM}}_{\text{asg}}^{\text{Pareto}}}-\text{ETC}. \end{array}$$

By referring to (23), the modification of generation capacity is performed in Step (c) consecutively with the load flow solution performed in Step (d) so that the ATC is determined by considering the firm transfer of CBM.

*Step (g).* Repeat Steps (a)–(f) to determine ATC for the next transfer case between the selling and buying areas. The determination of ATC for the next transfer case will also consider the same CBMs determined for the assisting and assisted areas.

**Figure 3:**
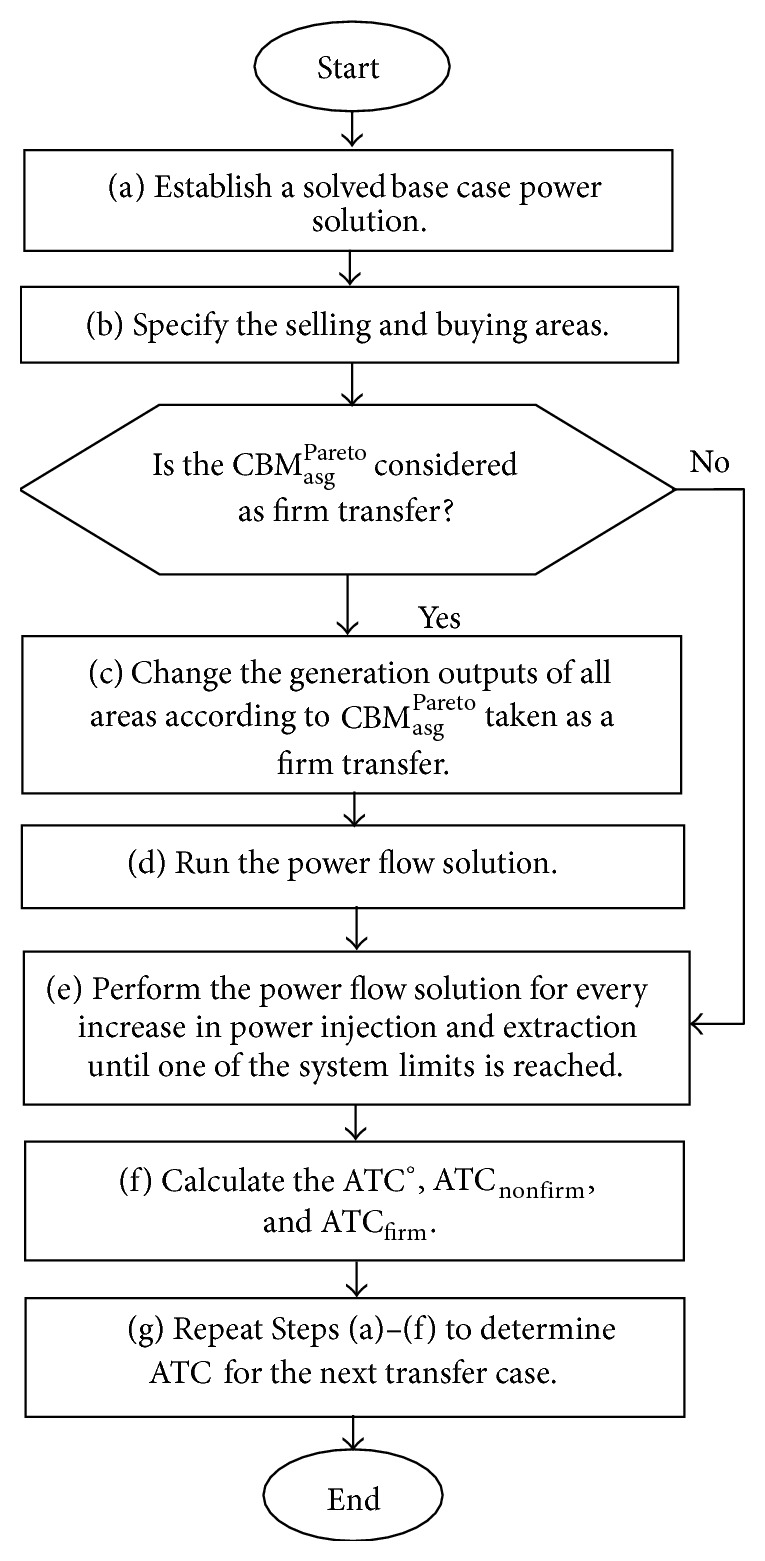
Flowchart of firm and nonfirm ATC determination technique.

The flowchart of ATC determination that takes into account the firm and nonfirm transfer margins of CBM_asg_^Pareto^ is illustrated in Figure 3.

## 5. Results and Discussion

A modified IEEE-RTS79 is used to demonstrate the effectiveness of the proposed method in determining the CBM for each area [19, 22]. The generating units and transmission line information are given in [19, 22]. In this paper, the specified value of LOLE limit, *ξ*, is assumed to be 2.4 hrs/yr.

### 5.1. Capacity Benefit Margin Considering Interconnected System Reliability

In the base case condition of a modified IEEE-RTS79, the total generation, total load, and LOLE associated with each area is presented in Table 1. Based on the predetermined LOLE, areas 2 and 3 are considered the assisting areas and area 1 is referred to as the assisted area.

Table 2 presents the results of CBM considering tie-line reliability and is determined using the basic methodology discussed in [19]. It is observed that 88 MW and 33 MW are the amount of CBM reserved for the transfer from assisting areas 2 and 3 to area 1, respectively, resulting in the LOLE value being below 2.4 hrs/yr. Hence, new generation capacities of 2156 MW, 1660 MW, and 751 MW are obtained for areas 1, 2, and 3, respectively.

### 5.2. Multiobjective Capacity Benefit Margins Result Determined by the Ranking Index in Pareto-Based Evolutionary Programming Technique

It is noteworthy that Table 1 has presented the total generation capacity and total load for every area at base case condition of IEEE-RTS79. In conjunction with this matter, the LOLE less than 2.4 hrs/yr implies that the assisting areas 2 and 3 have sufficient amount of total reserve generation capacity that can be used as a reference to estimate the amount of CBM for accommodating the generation deficiency which may occur in the assisted area 1 with LOLE above 2.4 hrs/yr. Hence, the EP optimization technique is used to perform simultaneous determination of CBM that can be transferred from the assisting areas 2 and 3 towards the assisted area 1.

In the EP optimization technique, there are 10 individuals in a population representing the *x*par_*m*,asg=1_ or CBMs for assisting area 2. The same situation goes to the next population representing the *x*par_*m*,asg=2_ or CBMs for assisting area 3. The initial process of EP optimization technique will randomly generate a uniform distribution of *x*par_*m*,asg_ using (1) based on the reserve generating capacity available in the assisting area. In particular, the initial population, *x*par_*m*,asg=1_, for assisting area 2 is obtained through the randomly generated variables that are in the range of 1 MW and 607 MW. This signifies that 1748 MW − 1141 MW = 607 MW is the reserved generating capacity available in the assisting area 2. In the overleaf case, that is, referring to the assisting area 3, the initial population, *x*par_*m*,asg=2_, is obtained via the randomly generated variables which are within the range of 1 MW and 200 MW. Both of the *x*par_*m*,asg_ representing the initial population for assisting area 2 and area 3 are tabulated in Table 3. Simultaneously, both of the initial populations are applied into the mutation in (7) and pairwise comparison process (10) to obtain *xoff*⁡_*m*,asg_ and a new *x*par_*m*,asg_, respectively, for the assisting areas 2 and 3. All of the optimization process embedded in the EP optimization technique is repeated until the difference between maximum fitness, *f*_max⁡_, and minimum fitness, *f*_min⁡_, for the assisted area 1 is equal or less than the specified *ε* = 0.1. In the last iteration of EP optimization process, the average value of *x*par_*m*,asg_ for both populations represents the optimum value of CBM for assisting areas 2 and 3. The *x*par_*m*,asg_ obtained at the final iteration of EP optimization process are shown in Table 4. In relation to each population of *x*par_*m*,asg_, it is obvious that a relatively similar value is obtained for all of the individuals, and the average value of *x*par_*m*,asg_ in (12) may yield to CBM specified for the assisting areas 2 and 3. This result is obtained only for one optimization run of EP technique. The EP optimization technique is executed for several times so that the Pareto optimal fronts of CBMs (CBM_asg_^Pareto^) are obtained which provides flexibility to the transmission provider in selecting optimum CBMs in tandem with the changes of economic, load-serving entity requirement or resource planner. The analysis of CBM_asg_^Pareto^ will be elucidated in the following discussion.

**Figure 4:**
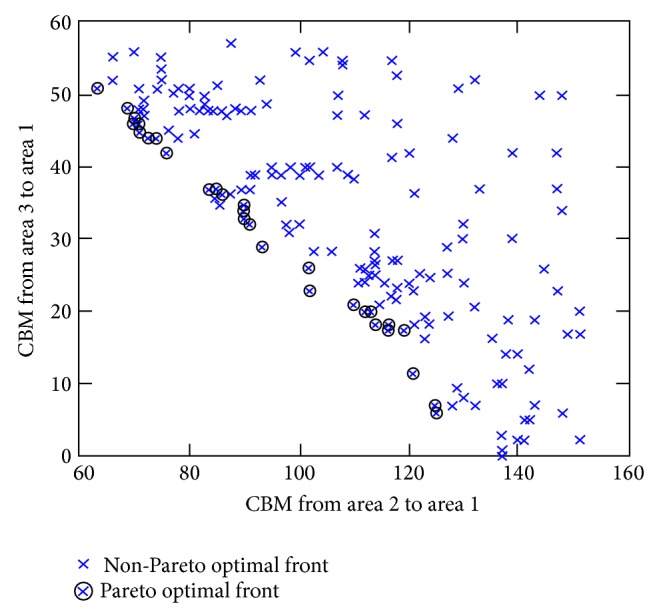
Pareto and non-Pareto optimal fronts of CBM for the two transfer cases.

Figure 4 shows different optimized values of CBM obtained at every execution of the EP optimization process. The *x*-axis represents the CBM transferred from the assisting area 2 to assisted area 1, whereas the *y*-axis represents the CBM transferred from assisting area 3 to assisted area 1.

**Figure 5:**
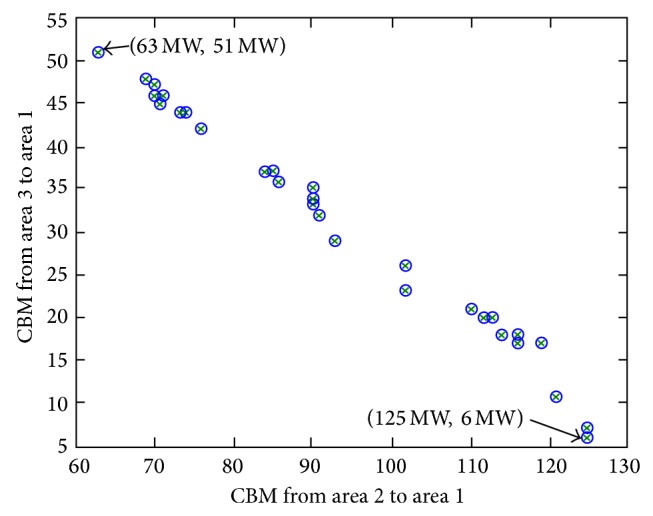
Pareto optimal fronts of CBMs for the two transfer cases.

It is observed that, with an increase in CBM associated with a particular assisting area, CBM at the other assisting area would decrease and vice versa. The best optimum values for the multiobjective function of CBMs are obtained based on the Pareto optimal front and the cluster for this case is illustrated in Figure 4. The other cluster represents the non-Pareto optimal front of  CBMs with excessive value which may yield to an invidious violation of power system security and ineffective utilization of the existing network resources.  Figure 5 represents the cluster of Pareto optimal front of CBMs extracted from Figure 4. In the Pareto optimal front, the results of CBM have less potential in violating system security compared with the excessive amount of CBMs obtained based on the non-Pareto optimal front.

Furthermore, the Pareto optimal front approach used in the EP technique gives sufficient flexibility to the ISO in selecting the optimum value of CBM for every transfer case depending on the system requirements. This is obviously contradictory with CBM results tabulated in Table 2 which are obtained using a basic approach [19]. Based on the CBM results shown in Table 2, ISO does not have the flexibility to select other choices with suitable set of CBMs for compensating any generation deficiency at different system operating states. In relation to Figure 5, CBM results for each area yielding to the Pareto optimal front are also tabulated in Table 5. Every result of Pareto optimal front CBM will be used as a reference to estimate the power transferred from assisting areas 2 and 3 to accommodate possible generation deficiency in the assisted area 1.

**Figure 6:**
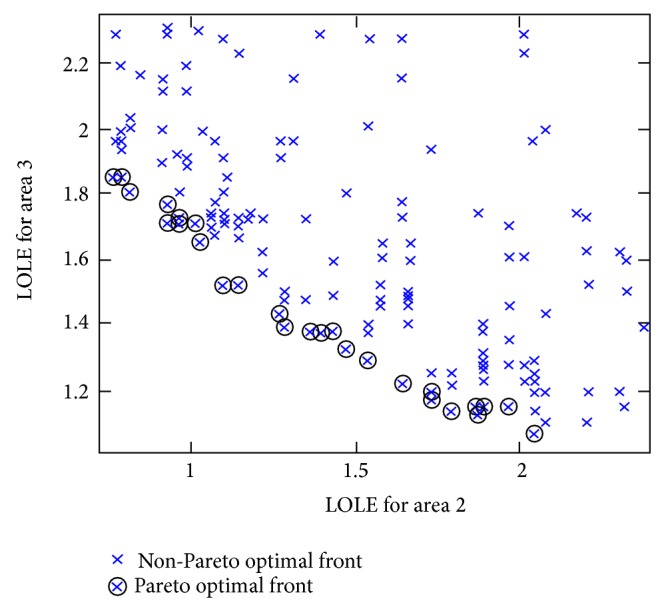
Pareto optimal fronts of LOLE for areas 2 and 3.

It is observed that the Pareto optimal front of CBM values was obtained while fulfilling the LOLE criterion of less than 2.4 hrs/yr. The other advantage of the proposed method is that CBM_asg_^Pareto^ results also yield Pareto optimal front clusters of LOLE and difference in LOLE values. This can be verified in Figure 6 where LOLE located at the Pareto optimal front cluster refers to the CBM_asg_^Pareto^ results obtained for each case of power transfer depicted in Figure 4. Consequently, the results of total LOLE obtained through (15) are arranged in ascending order and the ranking index is assigned to every result to distinguish the reliability of the assisting areas shown in Table 6. With respect to each value of total LOLE, total CBM_asg_^Pareto^ was obtained based on the two transfer cases also shown in Table 6. The total CBM_asg_^Pareto^ is equivalent to CBM for an assisted area, CBM_asd=1_.

**Figure 7:**
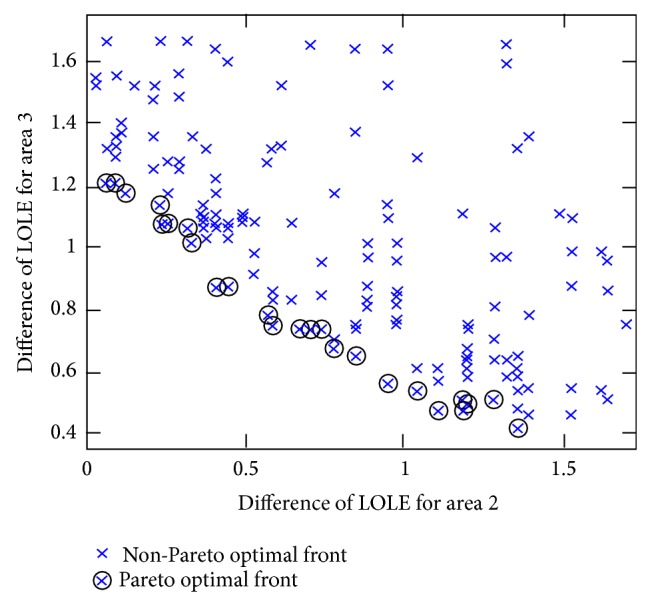
Pareto optimal fronts of LOLE difference for areas 2 and 3.

Figure 7 represents the difference between LOLE values located at the Pareto optimal front cluster. The results are referring to the CBM_asg_^Pareto^ obtained based on the power transfer cases shown in Figure 4. Then, the results of total LOLE difference calculated using (17) were arranged in ascending order and the ranking index is assigned to each result indicating level of reliability available for the assisting areas as shown in Table 6. Table 6 reveals that the total CBM_asg_^Pareto^ or CBM_asd_^Pareto^ values were arranged according to the total LOLE and total LOLE difference possessing the same ranking index. The results divulge that the Pareto-based EP method has the advantage of providing simultaneous optimum results of CBM_asg_^Pareto^, LOLE, and difference of LOLE in which all are located at the Pareto optimal front cluster.

As noted earlier and clearly presented in Table 6, the proposed method has the advantage of providing several choices of CBM that can be selected by ISO based on the ranking index of total LOLE and/or total LOLE difference. For instance, the ISO shall set the CBM_asg_^Pareto^, respectively, to 125 MW and 6 MW for the transfer case from assisting areas 2 and 3, respectively, to the assisted area 1 so that the assisting areas will operate in a highly reliable condition because the aforementioned power transfers are obtained based on the lowest total LOLE of 2.608 hrs/yr at the 1st ranking index. The combination of CBM_asg_^Pareto^ for both transfer cases will provide a total CBM_asg_^Pareto^ of 131 MW which results in the lowest total LOLE difference of 1.278 hrs/yr at the 1st ranking index as shown in Table 6. Due to a relatively large total CBM_asg_^Pareto^ of 131 MW, the tie-line capacity will not be fully utilized as a medium power transfer based ATC for electricity transfer. The total CBM_asg_^Pareto^ of 131 MW can also be obtained at the 10th ranking index as shown in Table 6. However, a total CBM_asg_^Pareto^ of 131 MW at the 10th ranking index will not be the best choice for the ISO since the assisting areas will operate in a less reliable condition due to the total LOLE of 2.673 hrs/yr and total LOLE difference of 1.343 hrs/yr. Furthermore, the 10th ranking index yields to a result that is close with the largest total CBM_asg_^Pareto^ of 136 MW located at the 12th ranking index. However, total LOLE of 2.694 hrs/yr and total LOLE difference of 1.364 hrs/yr signify a reasonable or moderately reliable operation of the assisting areas in conjunction with the largest total CBM_asg_^Pareto^ of 136 MW at the 12th ranking index.

In another situation whereby the ISO is not interested in a highly reliable condition of a power system, the CBM_asg_^Pareto^ of 70 MW can be selected for the transfer case from assisting area 2 to area 1 and the CBM_asg_^Pareto^ of 47 MW can be chosen for the transfer case from assisting area 3 to assisted area 1. This would be a less reliable choice prior to the largest value of total LOLE which is 3.115 hrs/yr at the 29th ranking index as tabulated in Table 6. Consequently, the total CBM_asg_^Pareto^ of 117 MW is obtained contributing to the largest total LOLE difference of 1.785 hrs/yr located at the 29th ranking index. For this case, a highly reliable condition incurred from a specific amount of CBM reserved through tie-line capacity is not the main intention for the ISO. Besides, ISO is more interested in the utilization of tie-line capacity for ATC in order to enhance and perform as an important role in the electricity market. Similar to the 29th ranking index, the CBM_asg_^Pareto^ of 117 MW at the 22nd ranking index can also be used in this case study. It has the advantage in providing total LOLE of 2.905 hrs/yr and total LOLE difference of 1.575 hrs/yr which is much better than the results obtained at the 29th ranking index. By comparing with the total CBM of 117 MW at the 22nd ranking index, ISO may choose the lowest value of total CBM, that is, 114 MW at the 28th ranking index, only when the objective is not solely on the reliability improvement of the assisting areas.

In a detailed analysis, ISO may select the CBM_asg_^Pareto^ of 90 MW and 33 MW for the transfer case from the assisting areas 2 and 3 to the assisted area 1, respectively, so that the assisting areas are operating at the mid ranking level (index 15) having the total LOLE of 2.734 hrs/yr and total LOLE difference of 1.405 hrs/yr. This indicates that ISO has chosen the value of CBM_asg_^Pareto^ for both transfer cases resulting in 50% priority on the reliability of assisting areas and 50% priority on the power transfer based ATC reserved for electricity market activities. The aforementioned discussion shows that the optimal value of CBM specified for each case of power transfer is actually dependent on similar ranking indices of total LOLE and total LOLE difference.

The previous results have well demonstrated that CBM_asg_^Pareto^, LOLEs, and difference of LOLEs clustered in the Pareto optimal front are the criteria to be satisfied by the ISO before conducting the finest selection of CBM_asg_^Pareto^. The performance of the Pareto optimal front embedded in the proposed optimization technique is not limited only to the CBM_asg_^Pareto^ value that provides the highest reliability of assisting areas due to the lowest total LOLE and total LOLE difference associated with the 1st ranking index. Nevertheless, it also is not confined to the CBM_asg_^Pareto^ value with large amount of ATC yielding to the largest total LOLE and total LOLE difference selected at the 29th ranking index. This implies that ISO has several choices for CBM for each case of power transfer depending on the ranking index selected based on the Pareto optimal front of total LOLE and total LOLE difference.

### 5.3. Performance Comparison with Existing Capacity Benefit Margin Calculation Methods

**Figure 8:**
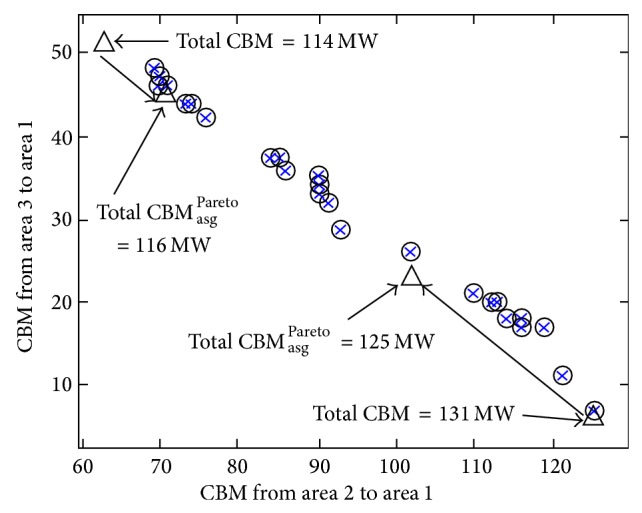
Total CBM_asg_^Pareto^ selection based on the ranking index in Pareto optimal front concept.

It is worthwhile to mention that the proposed method is robust in providing simultaneous optimum results of the CBM_asg_^Pareto^, LOLE, and difference in LOLE, all of which are located at the Pareto optimal front cluster. In the proposed method, the ranking index has the advantage of providing a clearer depiction on the relationship between the three optimal results which will be a great help to the ISO in making the finest decision for selecting optimum CBM values. This is contradictory to other methods in [17], whereby the optimization process is performed separately to find the minimum total LOLE, minimum total LOLE difference, or minimum CBM considering weight of the tie-lines. As shown in Figure 8, the lowest total LOLE of 2.608 hrs/yr and lowest total LOLE difference of 1.278 hrs/yr, computed using the method presented in [17], will give a total CBM result of 131 MW which is quite large according to the Pareto optimal front tabulated in Table 6. Using both methods discussed in [17], ISO does not have a choice other than to utilize a large total CBM value of 131 MW to ensure a highly reliable operating condition of the assisting areas in accordance with the lowest total LOLE of 2.608 hrs/yr and lowest total LOLE difference of 1.278 hrs/yr. Thus, the proposed method of Pareto optimal front provides a solution to the abovementioned problem by providing the total CBM_asg_^Pareto^ of 125 MW at the 2nd ranking index considered as the other option that is smaller than the total CBM of 131 MW at the 1st ranking index in Table 6 and Figure 8. The result of total CBM_asg_^Pareto^, that is, 125 MW at the 2nd ranking index, also provides a highly reliable operating condition for the assisting areas that are nearly identical with the lowest total LOLE and lowest total LOLE difference at the 1st ranking index. Consequently, the total CBM_asg_^Pareto^ of 125 MW at the 2nd ranking index provides a more conservative space to transfer the power based ATC compared with the total CBM of 131 MW at the 1st ranking index.

The method discussed in [17] provides some limited choices of CBM results as it considers the weight specified on each tie-line. However, the proposed method provides several CBM_asg_^Pareto^ values without considering the weight for the tie-lines. Once the tie-line weight is not considered in [17], the minimum total CBM of 114 MW is obtained. It is depicted in Figure 8 and Table 6 that the total CBM of 114 MW is obtained at the assisted area when the CBMs of 63 MW and 51 MW are transferred from the assisting areas 2 and 3, respectively. However, the total LOLE of 3.113 hrs/yr and total LOLE difference of 1.783 hrs/yr are relatively large at the 28th ranking index although the minimum total CBM of 114 MW is obtained using the abovementioned equation given in [17]. Therefore, the proposed Pareto optimal front concept is used to provide several choices of solution that are relatively similar to the minimum total CBM of 114 MW. For this case, the total CBM_asg_^Pareto^ of 116 MW is chosen from the 23rd ranking index of Pareto optimal front and it is the nearest value to the minimum total CBM of 114 MW. By referring to Figure 8 and Table 6, it can be observed that the total CBM_asg_^Pareto^ of 116 MW will improve the reliability of the assisting areas due to the total LOLE of 2.912 hrs/yr and total LOLE difference of 1.583 hrs/yr which are smaller than the LOLE results obtained from the minimum total CBM of 114 MW. For other cases of different weights assigned to each tie-line, the selection of CBM result is performed similarly by referring to the abovementioned explanation of Pareto optimal front concept.

### 5.4. Results of Available Transfer Capability Incorporating Capacity Benefit Margin

In this section, the ATC results are obtained based on the four cases of power transfer as shown in Tables 7, 8, 9, and 10. The results of ATCs are obtained by considering the firm and nonfirm transfers of CBM_asg_^Pareto^ located at the Pareto optimal front cluster as depicted in Table 6. Hence, for every case of power transfer, there are 29 results of ATC that give the flexibility to the ISO in choosing a suitable power transfer. It is noted that CBM_asg_^Pareto^ taken as a firm transfer contributes to slightly larger ATC values as compared to CBM_asg_^Pareto^ which is taken as a nonfirm transfer. It can be concluded that, by incorporating the nonfirm transfer of CBM_asg_^Pareto^ into ATC, there will be loss for certain amount of ATC in the power transfer contracts.

## 6. Conclusion

This paper has presented a new approach for calculating CBM taking into account tie-line reliability in the interconnected system. The proposed approach employs the ranking index in a Pareto-based EP technique that provides several choices of optimum CBM values. The effectiveness of the proposed method in determining the CBM has been tested on the modified IEEE-RTS79. The results presented have shown that the Pareto optimal front of CBMs is an inexpensive solution compared to the CBMs located at the non-Pareto optimal front. The other advantage associated with the proposed method is due to its ability in providing simultaneous optimal results of CBM, LOLE, and LOLE difference whereby all are located at the Pareto optimal front cluster. Hence, selection of the result does not rely solely on the value of CBM, but it is also concurrently based on the impact of total LOLE and total LOLE difference included under the ranking of Pareto optimal front. In short, ISO has the flexibility to select the CBM at the Pareto optimal front referring to the ranking index of total LOLE and total difference of LOLE. Finally, CBM taken as a firm transfer yields to a relatively large value of ATC compared to CBM considered as nonfirm transfer.

## Figures and Tables

**Table 1:** Generation, load, and LOLE for the three areas.

Area	Generation [MW]	Load [MW]	LOLE [hrs/yr]
1	2035	1125	4.7756
2	1748	1141	0.6380
3	784	584	0.6917

**Table 2:** CBM results considering interconnected system reliability using the method introduced in [19].

Area	Generation [MW]	CBM [MW]	LOLE [hrs/yr]
Assisted area 1	2156	121	2.3972
Assisting area 2	1660	88	1.3943
Assisting area 3	751	33	1.3569

**Table 3:** Initial population of EP technique for the assisting areas 2 and 3.

Number of individuals	Assisting area 2		Assisting area 3	
	*x*par_*m*,asg=1_ or CBM (MW)	LOLE (hrs/yr)	*x*par_*m*,asg=2_ or CBM (MW)	LOLE (hrs/yr)
1	474.61	52.67	47.96	1.89
2	237.57	5.22	71.63	3.44
3	147.71	2.29	165.24	37.63
4	246.18	5.46	4.08	0.70
5	59.55	1.03	9.60	0.80
6	81.11	1.25	34.80	1.43
7	572.83	137.47	130.82	15.57
8	581.37	148.06	147.35	23.20
9	350.15	15.24	130.55	15.57
10	37.29	0.85	91.19	5.62

**Table 4:** Final population for the assisting areas 2 and 3 based on one run of EP optimization process.

Number of individuals	Assisting area 2		Assisting area 3	
	*x*par_*m*,asg=1_ or CBM (MW)	LOLE (hrs/yr)	*x*par_*m*,asg=2_ or CBM (MW)	LOLE (hrs/yr)
1	86	1.32	35	1.43
2	86	1.32	35	1.43
3	86	1.32	35	1.43
4	86	1.32	35	1.43
5	86	1.32	35	1.43
6	86	1.32	35	1.43
7	86	1.32	35	1.43
8	86	1.32	35	1.43
9	85	1.29	35	1.43
10	85	1.29	35	1.43

**Table 5:** Pareto optimal front of CBM values for each area.

EP run	Assisted area 1		Assisting area 2		Assisting area 3	
	CBM [MW]	LOLE [hrs/yr]	CBM [MW]	LOLE [hrs/yr]	CBM [MW]	LOLE [hrs/yr]
1	114	2.3997	63	1.0646	51	2.0485
2	117	2.3627	69	1.1475	48	1.8874
3	117	2.3794	73	1.1739	44	1.731
4	121	2.3967	85	1.2913	36	1.4705
5	117	2.3656	70	1.1458	47	1.9693
6	117	2.3723	71	1.1237	46	1.8714
7	116	2.3867	70	1.1458	46	1.8714
8	116	2.3941	71	1.1237	45	1.7887
9	118	2.3641	74	1.1847	44	1.731
10	118	2.3667	76	1.2076	42	1.6394
11	121	2.3996	86	1.3183	35	1.431
12	122	2.3785	86	1.3183	36	1.4705
13	125	2.3411	90	1.3775	35	1.431
14	121	2.3954	84	1.2902	37	1.539
15	122	2.3766	85	1.2913	37	1.539
16	124	2.3534	90	1.3775	34	1.3913
17	123	2.3695	90	1.3775	33	1.3569
18	123	2.3711	91	1.3914	32	1.2769
19	122	2.3931	93	1.428	29	1.2597
20	128	2.3248	102	1.5166	26	1.1351
21	125	2.3697	102	1.5166	23	1.0929
22	131	2.3091	110	1.6479	21	1.0247
23	132	2.3999	112	1.7002	20	1.0107
24	133	2.3934	113	1.7095	20	1.0107
25	132	2.3985	114	1.7198	18	0.9511
26	134	2.373	116	1.7116	18	0.9511
27	133	2.3841	116	1.7116	17	0.9235
28	136	2.3455	119	1.7702	17	0.9235
29	132	2.3932	121	1.8074	11	0.8044

**Table 6:** CBM results with ranking index of total LOLE and total LOLE difference.

CBM_asd_^Pareto^ received by area 1 [MW]	CBM_asg_^Pareto^ from area 2 to area 1 [MW]	CBM_asg_^Pareto^ from area 3 to area 1 [MW]	Total LOLE [hrs/yr]	Total difference of LOLE [hrs/yr]	Rank index
131	125	6	2.608	1.278	1
125	102	23	2.610	1.280	2
132	121	11	2.612	1.282	3
132	125	7	2.629	1.300	4
133	116	17	2.635	1.305	5
128	102	26	2.652	1.322	6
134	116	18	2.663	1.333	7
123	91	32	2.668	1.339	8
132	114	18	2.671	1.341	9
131	110	21	2.673	1.343	10
122	93	29	2.688	1.358	11
136	119	17	2.694	1.364	12
132	112	20	2.711	1.381	13
133	113	20	2.720	1.391	14
123	90	33	2.734	1.405	15
124	90	34	2.769	1.439	16
122	86	36	2.789	1.459	17
125	90	35	2.809	1.479	18
121	84	37	2.829	1.500	19
122	85	37	2.830	1.501	20
118	76	42	2.847	1.517	21
117	73	44	2.905	1.575	22
116	71	45	2.912	1.583	23
118	74	44	2.916	1.586	24
117	71	46	2.995	1.665	25
116	70	46	3.017	1.688	26
117	69	48	3.035	1.705	27
114	63	51	3.113	1.783	28
117	70	47	3.115	1.785	29

**Table 7:** Results of ATC from area 1 to area 2.

EP run	ATC_base_ [MW]	ATC_firm_ [MW]	ATC_nonfirm_ [MW]
1	586	542	523
2	586	538	517
3	586	535	513
4	586	527	501
5	586	537	516
6	586	537	515
7	586	537	516
8	586	537	515
9	586	535	512
10	586	533	510
11	586	526	500
12	586	526	500
13	586	524	496
14	586	528	502
15	586	527	501
16	586	524	496
17	586	524	496
18	586	523	495
19	586	522	493
20	586	515	484
21	586	515	484
22	586	510	476
23	586	508	474
24	586	508	473
25	586	507	472
26	586	506	470
27	586	506	470
28	586	504	467
29	586	502	465

**Table 8:** Results of ATC from area 1 to area 3.

EP run	ATC_base_ [MW]	ATC_firm_ [MW]	ATC_nonfirm_ [MW]
1	271	258	220
2	271	259	223
3	271	260	227
4	271	262	235
5	271	259	224
6	271	259	225
7	271	259	225
8	271	260	226
9	271	260	227
10	271	260	229
11	271	262	236
12	271	262	235
13	271	262	236
14	271	262	234
15	271	262	234
16	271	263	237
17	271	263	238
18	271	263	239
19	271	264	242
20	271	265	245
21	271	265	248
22	271	266	250
23	271	266	251
24	271	266	251
25	271	267	253
26	271	267	253
27	271	267	254
28	271	267	254
29	271	268	260

**Table 9:** Results of ATC from area 2 to area 1.

EP run	ATC_base_ [MW]	ATC_firm_ [MW]	ATC_nonfirm_ [MW]
1	1171	1115	1108
2	1171	1110	1102
3	1171	1106	1098
4	1171	1096	1086
5	1171	1109	1101
6	1171	1108	1100
7	1171	1109	1101
8	1171	1108	1100
9	1171	1106	1097
10	1171	1104	1095
11	1171	1095	1085
12	1171	1095	1085
13	1171	1091	1081
14	1171	1097	1087
15	1171	1096	1086
16	1171	1091	1081
17	1171	1091	1081
18	1171	1090	1080
19	1171	1088	1078
20	1171	1080	1069
21	1171	1080	1069
22	1171	1073	1061
23	1171	1071	1059
24	1171	1070	1058
25	1171	1069	1057
26	1171	1067	1055
27	1171	1067	1055
28	1171	1065	1052
29	1171	1063	1050

**Table 10:** Results of ATC from area 3 to area 1.

EP run	ATC_base_ [MW]	ATC_firm_ [MW]	ATC_nonfirm_ [MW]
1	71	21.5	20
2	71	24.5	23
3	71	28.5	27
4	71	36.5	35
5	71	25.5	24
6	71	26.5	25
7	71	26.5	25
8	71	27.5	26
9	71	28.5	27
10	71	30.5	29
11	71	37.5	36
12	71	36.5	35
13	71	37.5	36
14	71	35.5	34
15	71	35.5	34
16	71	38.5	37
17	71	39.5	38
18	71	40.5	39
19	71	43.5	42
20	71	46.5	45
21	71	49.5	48
22	71	51.5	50
23	71	52.5	51
24	71	52.5	51
25	71	54.5	53
26	71	54.5	53
27	71	55.5	54
28	71	55.5	54
29	71	21.5	20
